# Nano-scale morphology of cardiomyocyte t-tubule/sarcoplasmic reticulum junctions revealed by ultra-rapid high-pressure freezing and electron tomography

**DOI:** 10.1016/j.yjmcc.2020.12.006

**Published:** 2021-04

**Authors:** E.A. Rog-Zielinska, R. Moss, W. Kaltenbacher, J. Greiner, P. Verkade, G. Seemann, P. Kohl, M.B. Cannell

**Affiliations:** a Institute for Experimental Cardiovascular Medicine, University Heart Center Freiburg·Bad Krozingen, Freiburg, Germany; b Faculty of Medicine, University of Freiburg, Freiburg, Germany; c School of Biochemistry, Faculty of Biomedical Sciences, University of Bristol, Bristol, UK; d School of Physiology, Pharmacology & Neuroscience, University of Bristol, Bristol, UK

## Abstract

Detailed knowledge of the ultrastructure of intracellular compartments is a prerequisite for our understanding of how cells function. In cardiac muscle cells, close apposition of transverse (t)-tubule (TT) and sarcoplasmic reticulum (SR) membranes supports stable high-gain excitation-contraction coupling. Here, the fine structure of this key intracellular element is examined in rabbit and mouse ventricular cardiomyocytes, using ultra-rapid high-pressure freezing (HPF, omitting aldehyde fixation) and electron microscopy. 3D electron tomograms were used to quantify the dimensions of TT, terminal *cisternae* of the SR, and the space between SR and TT membranes (dyadic cleft). In comparison to conventional aldehyde-based chemical sample fixation, HPF-preserved samples of both species show considerably more voluminous SR terminal *cisternae*, both in absolute dimensions and in terms of junctional SR to TT volume ratio. In rabbit cardiomyocytes, the average dyadic cleft surface area of HPF and chemically fixed myocytes did not differ, but cleft volume was significantly smaller in HPF samples than in conventionally fixed tissue; in murine cardiomyocytes, the dyadic cleft surface area was higher in HPF samples with no difference in cleft volume. In both species, the apposition of the TT and SR membranes in the dyad was more likely to be closer than 10 nm in HPF samples compared to CFD, presumably resulting from avoidance of sample shrinkage associated with conventional fixation techniques. Overall, we provide a note of caution regarding quantitative interpretation of chemically-fixed ultrastructures, and offer novel insight into cardiac TT and SR ultrastructure with relevance for our understanding of cardiac physiology.

## Highlights

• The cardiac dyadic cleft, where T-tubules and sarcoplasmic reticulum are closely opposed, is key to cardiac excitation-contraction coupling.
• Most knowledge about the ultrastructure of the dyad comes from electron microscopic studies, traditionally involving chemical fixation.
• By using ultra-fast vitrification, we demonstrate that the dyadic cleft has dramatic qualitative and quantitative differences in morphology.

## Graphical abstract

**Figure:**
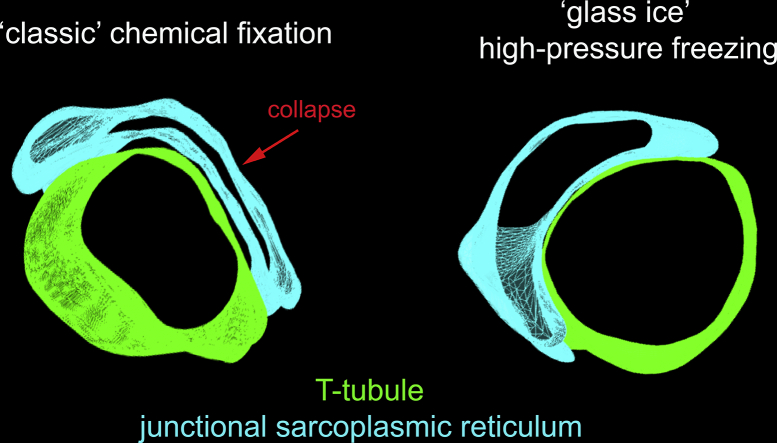

## 1 Introduction

The link between electrical excitation and Ca^2+^ release from the sarcoplasmic reticulum (SR) is a key process in cardiac excitation-contraction coupling (ECC) [1]. ECC involves the entry of Ca^2+^, primarily *via* L-type Ca^2+^ channels, and diffusion of that Ca^2+^ to the surface of the SR where specialized SR Ca^2+^ release channels, called ryanodine receptors (RyR), are located. In cardiac myocytes, invaginations of the surface membrane in form of transverse tubules (TT) carry excitation deep into the cell interior, allowing for near-synchronous Ca^2+^ release from closely opposed SR throughout the cell. In the TT–SR membrane complex, called the dyad, precise control of ECC allows for stable amplification of Ca^2+^ influx to levels that govern efficient activation of the contractile machinery [2,3].

Conceptual and computational models of cardiac ECC and Ca^2+^ transport/ storage processes require a detailed understanding of the nanoscopic structure of the dyad and of the distribution of Ca^2+^ binding proteins [4]. Previous ultra-structural studies of the membrane systems underlying ECC have been based almost entirely on the analysis of two-dimensional (2D) transmission electron microscopy (EM) images. More recently, several studies have reported 3D data obtained by serial block-face scanning EM and electron tomography (ET) and reconstruction [[5], [6], [7], [8], [9], [10], [11], [12], [13]].

Most of the available structural data have been obtained using murine models, though there is concern as to the extent to which mice and rats mimic human myocardial structure and function. To that end, lagomorphs may offer a more relevant model for translational cardiac research. This ranges from responsiveness to pathological processes (such as myocardial ischaemia) and pharmacological interventions, to ion channel and transporter distribution, features of the action potential, mechanisms of Ca^2+^ homeostasis, and appearance of the TT network [[14], [15], [16], [17]]. At this time however, rabbit cardiomyocyte dyad ultrastructure remains relatively unexplored at the nanometre scale.

TT and their axial projections form a complex network of tubular membrane invaginations with diameters ranging from 20 to 450 nm [18]. The SR is a structurally distinct intracellular membranous network with Ca^2+^ storage and protein processing functions [1]. In terms of Ca^2+^ signalling, the SR can be split into two regions with separable functions: (i) junctional SR (jSR) formed by terminal *cisternae* that partially wrap around TT, and (ii) network SR (nSR) that forms an extensive mesh-like structure between *Z*-lines [19]. It is generally accepted that the jSR supports Ca^2+^-induced Ca^2+^-release (CICR) *via* RyR, while the nSR enables re-uptake of released Ca^2+^, ending cell contraction [1,20].

In the dyad, TT and jSR membranes are thought to approach each other to within 10–20 nm [[21], [22], [23]]. The most important EM features of the dyad are: (1) close apposition of TT and jSR membranes; (2) electron-dense material in the lumen of the SR, which includes the Ca^2+^-binding protein calsequestrin; and (3) electron-dense globular elements bridging the dyadic cleft between the TT and jSR membranes, assumed to reflect RyR and associated proteins [24].

The spatial organisation of the dyad is essential, not only for bringing together the proteins that underlie CICR, but also for concentrating and separating the local dynamic fluctuations in Ca^2+^ concentration in the dyadic cleft from global (‘cell-wide’) Ca^2+^ changes [25]. This organisation can be quantitatively explored in 3D using ET. However, the quality and reliability of structural EM data is heavily dependent on sample quality and sample preparation. Of the many steps involved in sample processing, initial preservation exerts the largest influence on final EM quality and data fidelity [26]. The conventional method for cardiac sample preservation involves coronary perfusion (or plunge/ ‘chunk’ fixation) with aldehyde-based solutions for chemical fixation and subsequent dehydration (CFD). This well-established method carries the risk of altering the structure of biological samples by causing shrinkage and distortion, as well as loss, destruction, and displacement of molecular structures – all of which may give rise to erroneous conclusions regarding ultrastructural organisation [[27], [28], [29], [30]]. Such concerns have been a driving force for improvements to initial sample preservation – for example using vitrification rather than chemical fixation. Vitrification preserves the tissue by turning water-containing samples into a non-crystalline amorphous solid by ultra-rapid high-pressure freezing (HPF) [30,31]. HPF helps one to avoid many of the structural alterations (*e.g.* collapse and distortion of membranous organelles) associated with CFD [27,32].

In this study, we compare TT and jSR geometry and their dyadic organisation in rabbit and mouse cardiac myocyte samples, preserved using either HPF or CFD. We report significant differences in the shapes, volume ratios, and spatial interrelations of the membrane systems that form the dyad. These findings should be taken into account in conceptual and quantitative models of cardiac ECC, to improve our understanding of this vital process.

## 2 Materials and methods

All procedures were performed in accordance with UK legislation, and all samples were obtained from excess tissue being prepared for other studies. Ventricular myocytes were enzymatically isolated from the hearts of rabbits (New Zealand White, male, 2.5 kg, *N* = 3) and mice (C56Bl/6, male, 25 g, N = 3), as described previously [33]. Cardiac tissue from rabbits (N = 3) and mice (N = 3) was obtained using established methods [18].

Immediately following enzymatic isolation, cells were supplemented with 10% bovine serum albumin to reduce swelling and alterations in membrane configuration, gently pelleted and immediately either (i) fixed chemically with isosmotic Karnovsky's fixative (3:1:1 mix of sodium cacodylate, paraformaldehyde, and glutaraldehyde; 300 mOsm; Solmedia Limited, Shrewsbury, UK); or (ii) cryoimmobilized by HPF using an EM PACT2 + RTS HPF system (Leica Microsystems, Vienna, Austria). A separate subset of samples was supplemented with additional 300 mM mannitol (~5 min prior to HPF) to elevate the osmolarity of the solution, to counter any possible cell swelling.

Chemically fixed samples were processed (including dehydration) to Epoxy resin (Agar Scientific, Stansted, UK) as described previously [8]. HPF samples were freeze-substituted in 1% osmium tetraoxide and 0.1% uranyl acetate in acetone, using an EM AFS2 freeze-substitution unit (Leica Microsystems, Vienna, Austria), and then embedded in the same Epon resin [18,21].

Ultrathin (80 nm) sections for transmission EM were imaged using a 120 kV FEI Tecnai 12 (FEI Company, now Thermo-Fisher Scientific, Eindhoven, The Netherlands) fitted with a TVIPS F214 digital camera. Thick sections (250–380 nm) for ET were imaged using a 200 kV Tecnai 20 fitted with an FEI Eagle 4 K × 4 K camera, or 300 kV Tecnai TF30 and 4 K × 4 K charge-coupled device camera (UltraScan; Gatan, Pleasanton, CA). Images from a double tilt series (with 90° in-plane sample rotation in-between series) were collected between ±65°. Tilt series were aligned, reconstructed, and combined using IMOD [34], yielding final isotropic voxel of [1.55 nm] [3]. All cells analysed were assessed for contractile state, and excluded if resting sarcomere length was below 1.7 μm to exclude non-physiologically contractured samples. Volumes of TT and SR, as well as area and distance of their apposition, were calculated for each tomogram using either IMOD or custom Python programs (available on request).

### 2.1 Statistical analysis

Data are presented as individual points and mean ± SEM, and were analysed using Student's *t*-test or one-way ANOVA with Bonferroni's *post-hoc* test. A *p*-value of <0.05 was considered indicative of a statistically significant difference between the means.

## 3 Results

### 3.1 Structural appearance of TT and jSR

**Fig. 1:**
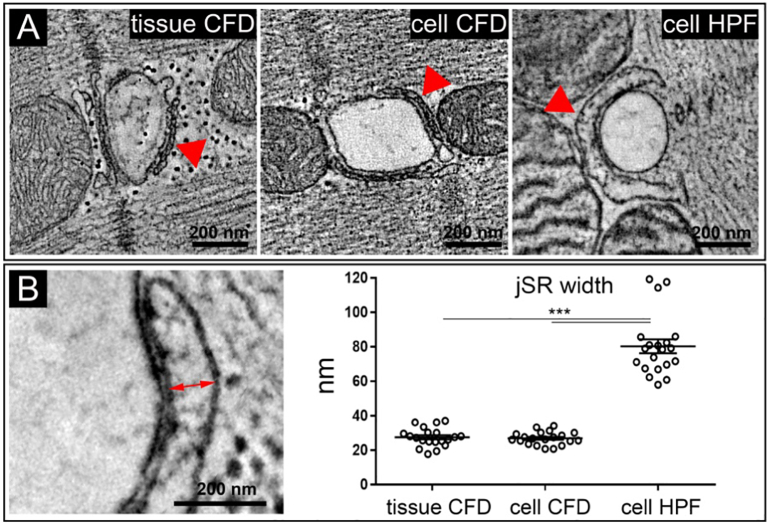
The jSR in rabbit ventricular myocytes is wider in HPF preserved cells, compared to CFD. A: Comparison of jSR (red arrows) in representative TT-perpendicular ET slices extracted from 3D volumes of rabbit ventricular (coronary perfusion-) chemically fixed and dehydrated tissue (tissue CFD), isolated CFD-preserved cells (cell CFD), and high-pressure frozen isolated cells (cell HPF, same batch as cell CFD). B: jSR width at its widest projection (red arrow) in rabbit cardiomyocytes is highest in cells preserved by HPF when compared both to isolated cells or tissue, preserved by CFD; ***p < 0.001; one-way ANOVA with Bonferroni's post-hoc test; *n* = 20 TT-jSR pairs/ 16–20 cells/ 3 hearts. (For interpretation of the references to colour in this figure legend, the reader is referred to the web version of this article.)

In prior EM studies, the cardiac jSR has been described as “flattened saccules or *cisternae*” [35] which are “relatively uniform in width” [36], and thus appearing as a “narrow sac” [37]. This view is consistent with the phenotype seen in our CFD samples, both of isolated cells and tissue (Fig. 1A). However, using HPF (omitting aldehyde-based fixatives and dehydration), the jSR of rabbit ventricular myocytes has a bulbous, distended appearance. To exclude potential artefacts associated with the lack of control over the plane of sectioning (which could affect apparent distances between membrane surfaces), we used ET to measure the width of jSR at their widest projection along an axis perpendicular to the associated TT (Fig. 1B). This analysis confirmed quantitatively that the jSR is significantly wider in HPF rabbit ventricular myocytes than in (same batch) CFD cell samples and in CFD tissue (Fig. 1B).

The extremely high speed of vitrification of the ~150–200 μm thick samples by HPF (<10 ms) should preclude any cellular volume changes, as the half-time of tissue swelling (*e.g.* in response to osmotic stress) is much slower (tens of seconds; see Fig. 1 in Moench et al.) [38]. In fact, even addition of 300 mM mannitol to the bath solution prior to HPF did not prevent the dilated jSR phenotype seenin HPF preserved samples (SR width 73.8 ± 4.7 nm and 70.7 ± 5.6 nm in control and mannitol-supplemented samples, respectively, Suppl. Fig. S1). We consider it highly unlikely, therefore, that fluid movement during HPF could explain the different appearance of jSR in HPF when compared to CFD samples.

**Fig. 2:**
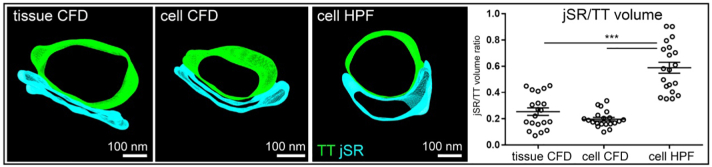
Partial jSR/TT volume ratio is higher in HPF *vs* CFD preserved rabbit ventricular cardiomyocytes. TT (green) and jSR (blue) were segmented and quantified in ET data of isolated cells preserved by HPF or CFD, and in (coronary perfusion-)CFD tissue; ***p < 0.001; one-way ANOVA with Bonferroni's post-hoc test; *n* = 20 TT-jSR pairs/ 16–20 cells/ 3 hearts. (For interpretation of the references to colour in this figure legend, the reader is referred to the web version of this article.)

Volumetric analysis of rabbit myocyte tomograms showed that local jSR volumes in HPF tomograms were higher when compared to CFD samples, whereas no differences in partial TT volumes between the sample groups were observed (Table 1). As a result, there was a markedly higher jSR/TT volume ratio in the HPF samples, compared to CFD (Fig. 2).

**Table 1:** TT, jSR, and dyad geometry (per 100 nm of TT length) in rabbit and mouse ventricular myocytes.

	TT volume (× 10^6^ nm^3^)	jSR volume (× 10^6^ nm^3^)	Dyad surface (× 10^3^ nm^2^)	Dyad volume (× 10^3^ nm^3^)
**Rabbit**				
Tissue CFD	4.29 ± 0.46	0.97 ± 0.11	45.9 ± 4.3	588 ± 58
Cells CFD	4.45 ± 0.48	0.81 ± 0.07	40.1 ± 3.2	400 ± 28^⁎⁎^
Cells HPF	3.97 ± 0.44	2.27 ± 0.22^⁎⁎⁎^†	42.3 ± 3.8	355 ± 30^⁎⁎⁎^

**Mouse**				
Tissue CFD	4.27 ± 0.46	1.2 ± 0.14	34.14 ± 3.4	270 ± 35
Cells HPF	4.09 ± 0.43	2.67 ± 0.17^⁎⁎⁎^	45.63 ± 4.9*	304 ± 32

Dyad defined as space of jSR-TT apposition of ≤20 nm; ***p < 0.001, **p < 0.01, *p < 0.05 *vs* tissue CFD; †p < 0.001 *vs* cells CFD; one-way ANOVA with Bonferroni's post-hoc test (rabbit), Student's *t*-test (mouse); *n* = 20 TT-jSR pairs/ 16–20 cells/ 3 hearts (rabbit); *n* = 21–23 TT-jSR pairs/ 14–21 cells/ 3 hearts (mouse).

**Fig. 3:**
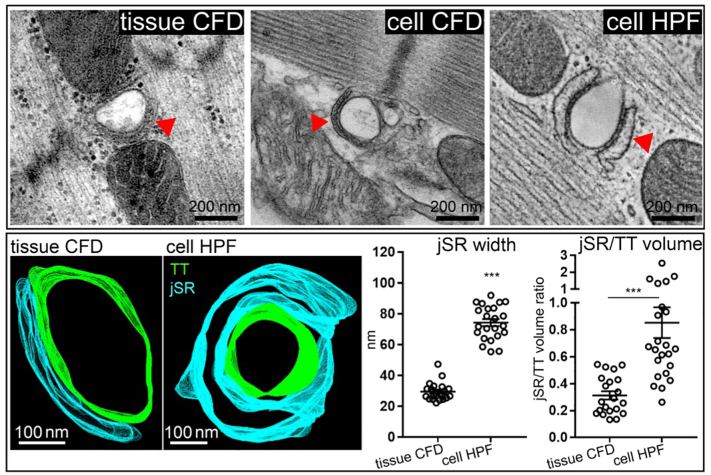
The jSR in mouse ventricular myocytes is wider and more voluminous upon HPF, compared to CFD. Top: Comparison of jSR (red arrows) in representative TT-perpendicular ET slices extracted from 3D volumes (tissue CFD and cell HPF) or 2D sections (cell CFD). Bottom: representative 3D models (green: TT; blue: jSR), segmented and quantified in ET data; ***p < 0.001; Student's *t*-test; *n* = 21–23 TT-jSR pairs/ 14–21 cells/ 3 hearts. (For interpretation of the references to colour in this figure legend, the reader is referred to the web version of this article.)

This difference in jSR appearance was not species-dependent, as similar effects of sample preparation on jSR phenotype and jSR/TT ratios were also observed in mouse ventricular tissue samples (Fig. 3, Table 1).

### 3.2 Dyadic cleft geometry

The structure of the rabbit dyadic cleft was also analysed in 3D, and cleft volumes and surface areas were measured. For all measurements, the dyad was defined as the space and surface area (of TT) within the region where the distance between TT and juxtaposed jSR membranes was ≤20 nm. Using this criterion, in rabbit ventricular myocytes the dyad surface area was comparable between groups (Table 1), while dyadic cleft volume was lower in HPF cells compared to CFD tissue (but not CFD cells). In mouse ventricular myocytes, dyadic surface was higher in HPF samples, with no change in dyad cleft volume, when compared to tissue CFD (Table 1).

**Fig. 4:**
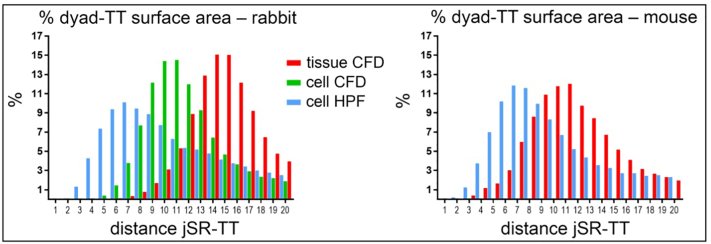
Dyadic geometry differences between HPF and CFD samples. Histograms showing the distribution (%) of the surface area associated with different dyadic spacing (minimum distance between TT and jSR) in ventricular tissue or cells from rabbit (left) and mouse (right), preserved by CFD or HPF. Dyad defined as space of jSR-TT apposition of ≤20 nm. Pooled data from *n* = 20 TT-jSR pairs/ 16–20 cells/ 3 hearts (rabbit); *n* = 21–23 TT-jSR pairs/ 14–21 cells/ 2–3 hearts (mouse).

Further examination of the distribution of the surface area associated with different dyadic spacing (based on distance between TT and jSR) revealed that in rabbit and mouse HPF-preserved samples the largest proportion of the dyadic area was associated with a jSR-TT spacing of ≤10 nm, whereas in CFD cells and tissue the majority of the dyadic area was associated with jSR-TT spacing of ≥10 nm (Fig. 4).

### 3.3 Ultrastructure of the jSR

**Fig. 5:**
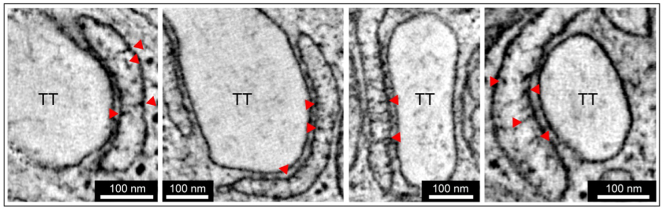
Structured electron densities within the lumen of jSR in rabbit isolated ventricular myocytes, preserved by HPF. Structures of interest (indicated with red arrows) were observed in virtually all HPF-preserved dyads; images representative of *n* = 20 TT-jSR pairs/ 16–20 cells/ 3 hearts. (For interpretation of the references to colour in this figure legend, the reader is referred to the web version of this article.)

HPF allowed us to obtain a more detailed view of the non-collapsed jSR lumen, revealing numerous local electron densities (Fig. 5). By analogy with the previous identification of the central electron density in CFD samples as calsequestrin [39], we suggest these electron densities could be polymerized calsequestrin [40]. Since calsequestrin may be linked to triadin and junctin [41], the close membrane association of the observed electron densities is plausible. These structured electron densities are not easily identified in CFD samples due to the collapse of jSR volumes (see *e.g.*Fig. 1).

## 4 Discussion

The extensive and intricate coupling of TT and SR membranes is fundamental for ECC and CICR in atrial and ventricular cardiac myocytes [1,42]. Changes in the microanatomy of the two membrane complexes and their spatial relationship can result in Ca^2+^ handling abnormalities and impaired contractile function [43].

Significant insight into TT to jSR coupling has been obtained using EM. However, sample preservation has conventionally involved CFD, which is known to introduce artefacts such as shrinkage and distortion of biological membrane compartments [[27], [28], [29], [30]]. Although HPF has been acknowledged to preserve biological samples more faithfully, the need for specialist equipment enabling ultra-fast heat extraction and pressure development for rapid vitrification of samples has hindered broader uptake of the technique. Even studies that have utilised HPF have also used chemically ‘pre-fixed’ samples, which does not entirely eliminate the likelihood of CFD artefacts [5,21,44].

Using HPF without aldehyde fixation, we find that the appearance of jSR membranes and dyadic clefts is qualitatively and quantitatively different from CFD samples. HPF results in the jSR appearing much less collapsed and the dyadic space exhibiting closer coupling of TT and SR membranes. Interestingly, in some tomograms it was also possible to identify the nSR. Fig. S2 illustrates the appearance of the nSR in rabbit ventricular myocytes after CFD and HPF. In general, HPF resulted in a smoother, move voluminous appearance to the nSR membrane outline with occasional small fenestrations similar to those described for skeletal muscle [45]. Encouragingly, no differences in TT volumes were observed between the different preservation approaches, suggesting that our findings regarding jSR size are not related to an overall sample expansion upon HPF.

We sought to assess the possibility that the different morphology we observed might arise from fluid movement such as might occur under an osmotic gradient that theoretically could arise locally upon gradual freezing of a saline-containing sample. In a subset of experiments using cells exposed to a hyperosmotic solution (two times normal tonicity) the jSR still maintained the bulbous, distended appearance, as seen in standard HPF conditions. This supports the idea that our observations are not simply an artefact caused by differential organelle swelling.

The jSR membranes are generally reported to approach their associated TT membrane to within 10–20 nm [12,[21], [22], [23]]. Here, we report dyadic gap widths that vary over a wider range of distances, to below 10 nm in HPF cells. It should be noted that using 3D ET data, we were able to assess the *actual* closest (surface-normal) distances between TT and jSR membrane surfaces. Thin section 2D EM data would be likely to overestimate the true dyadic gap width, due to cosine errors when not projecting along the shortest distance between two near-parallel surfaces. While there was a clear quantitative difference between HPF-preserved cells and CFD-preserved cells and tissues, we also noted differences between the latter two preparations. While the cause for this is currently unclear, it is possible that differences in storage time in fixative may have affected the CFD phenotype seen here (cells ~1 h, tissue >24 h). To avoid this, exposure to the shortest possible fixation time is recommended for CFD samples.

The variability in TT-jSR membrane distance observed here does not preclude accommodation of RyR, whose cytoplasmic component is thought to extend around 12 nm beyond the SR membrane. Recent molecular imaging results have shown that the in-plane arrangement of RyR is highly non-uniform, with small and larger clusters of RyR whose topology is susceptible to changes in cellular environment [[46], [47], [48], [49], [50], [51]]. Our observations are consistent with such a dynamic arrangement, and in regions of the dyad lacking RyR, the two membranes may either be pulled closer together (aided, potentially, by junctophilin), or be less well linked and more separated. In regions rich in RyR, TT and jSR membranes will then be forced apart by the RyR cytoplasmic domains, presumably supporting the 10–15 nm dyadic gap width [52], which is well represented in both HPF and CFD samples.

The dyadic cleft volume itself is a major factor determining local dyadic Ca^2+^ concentration changes during ECC. This, in turn, governs the probability of RyR opening. From consideration of diffusion in heterogeneous media [53], we estimate that a ~ 50% reduction in total free area for diffusion (*e.g.* by regions occupied by RyR or close apposition of TT and jSR membranes) could reduce the local diffusion coefficient by one third of that present in the absence of these additional restrictions. Furthermore, local constrictions in the dyadic space may serve to constrain Ca^2+^ level fluctuations, and possibly insulate other cellular Ca^2+^-sensitive pathways from unwanted ‘collateral’ activation by released Ca^2+^. Additionally, the geometric constrains imposed by the dyadic architecture may enable greater and/or more stable amplification as, for any given SR release flux, the local Ca^2+^ levels in the dyad will be higher. This would strengthen regenerative release, until local jSR depletion and induction decay terminates CICR [54].

Contemporary computational models of Ca^2+^ signalling in ECC of cardiomyocytes assume a simplified architecture for dyads, with a constant distance (10–20 nm) between jSR and TT membranes and a flat ‘low volume’ jSR morphology (“flattened cisternal extensions”) [54]. If models were to adopt an increase in the local jSR volume, this could slightly increase the amount of local Ca^2+^ available for release from the SR. A more significant effect might arise from an increase in the availability of free Ca^2+^ within the jSR, which is critical for the induction phase of CICR, because local jSR depletion will inhibit the regeneration inherent in CICR [54]. In connection with this and as noted above, while dyadic cleft volume is predicted to have little effect on spark amplitude or duration *per se*, it may significantly influence the probability that the opening of an individual RyR will lead to additional RyR opening, and thus alter the likelihood of triggering sparks [55]. Our results will therefore be useful in advancing the further development of geometrically realistic cardiac ECC models [4], including beat-by-beat changes to the topology of the dyads [13].

In summary, we present novel insight into the 3D ultrastructure of jSR and dyadic space, made possible by the use of HPF as an alternative to classic CFD sample preservation methods and 3D ET for nanometre-accurate reconstruction. Although not explored here, other intracellular elements – *e.g.* mitochondria – are likely to also exhibit systematic differences in morphology when using HPF instead of CFD. Further research is needed to explore the quantitative morphology of cellular membrane compartments, and their relevance for cardiomyocyte function.

## Appendix A Supplementary data

Supplementary material Image 1
