# Supplementary material for: Nano-scale morphology of cardiomyocyte t-tubule/sarcoplasmic reticulum junctions revealed by ultra-rapid high-pressure freezing and electron tomography
Source: J Mol Cell Cardiol. 2021 Apr;153:86–92. doi: 10.1016/j.yjmcc.2020.12.006 (PMC8035077; doi:10.1016/j.yjmcc.2020.12.006)
Supplement: Supplementary file 1 — Supplementary material [file mmc1.docx]

**Supplemental Figures**


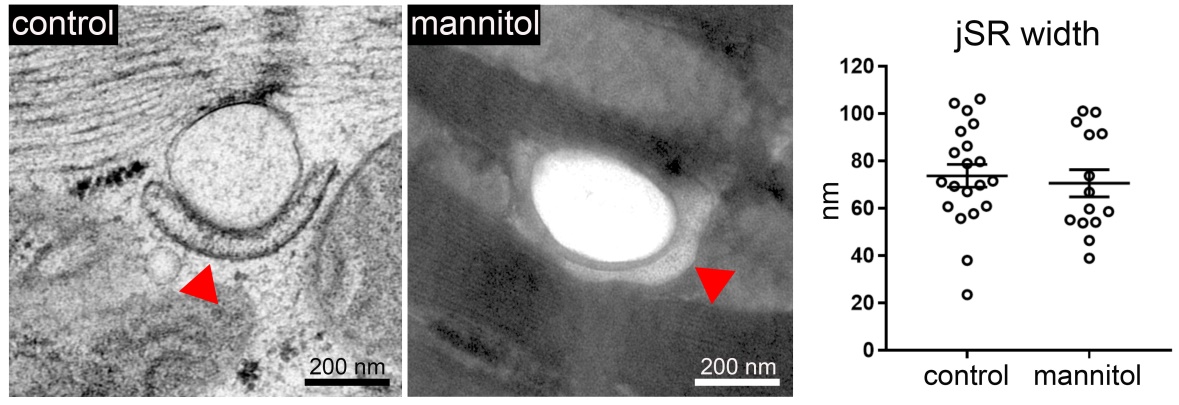


***Supplemental Figure 1. The distended structure of jSR in HPF-preserved rabbit ventricular myocytes is maintained under hyperosmotic conditions (addition of 300 mM mannitol).*** *Representative 2D images, n=14-20 jSR-TT pairs/ 14-20 cells/ 1 heart.*


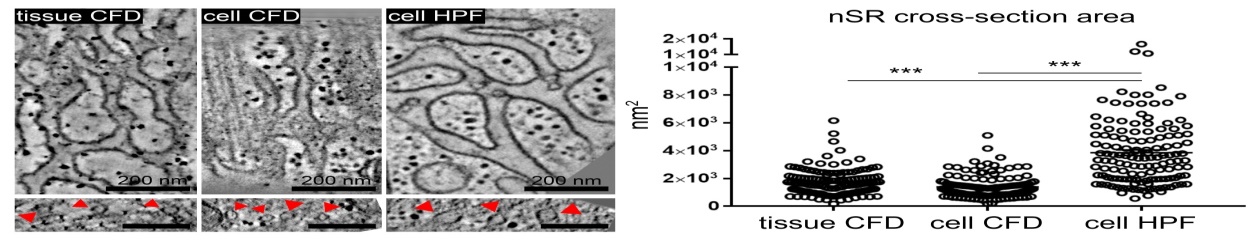


***Supplemental Figure 2. The cross-section of tubular elements of nSR network is higher in HPF vs CFD samples in rabbit ventricular myocytes****. Left: representative ET slices extracted from 3D volumes demonstrating the nSR network (bottom panel – cross-section, red arrowheads indicate the nSR elements in cross-section); ***p<0.001; one-way ANOVA with Bonferroni’s post-hoc test; n=135 nSR elements/ 16-20 cells/ 3 hearts.*
